# Identification of lipid metabolism-associated genes as prognostic biomarkers based on the immune microenvironment in hepatocellular carcinoma

**DOI:** 10.3389/fcell.2022.883059

**Published:** 2022-10-18

**Authors:** Xiangqian Gu, Chenshan Jiang, Jianguo Zhao, Qian Qiao, Mingyu Wu, Bing Cai

**Affiliations:** Department of Hepatobiliary Surgery, Wuxi People’s Hospital Affiliated Nanjing Medical University, Wuxi, China

**Keywords:** hepatocellular carcinoma, lipid metabolism, immune cell infiltration, immune checkpoint, prognosis

## Abstract

Lipid metabolism has been associated with progression of various cancers. However, the underlying mechanisms of the impact of lipid metabolism-associated genes (LMAGs) on the tumor immune microenvironment have not been well-elucidated. This study aimed to determine the effects of lipid metabolism on the progression and development of hepatocellular carcinoma (HCC). Expression profiles and clinical data of 371 and 231 patients with HCC were obtained from the TCGA and Internal Cancer Genome Consortium (ICGC) databases, respectively. Using Cox regression and LASSO regression analyses, a prognostic risk model was constructed based on the LMAG data. The tumor mutation burden (TMB), immune cell infiltration levels, and immune response checkpoints of the identified risk groups were determined and compared. A total of two clusters were identified based on the LMAG expression, showing significant differences in tumor stage and immune cell infiltration. A prognostic risk model based on four LMAGs was constructed and proven to have a significant prognostic value. The 1-, 3-, and 5-year survival rates in the high-risk group were 62.2%, 20.5%, and 8.1%, respectively, whereas those in the low-risk group were 78.9%, 28.1%, and 13.5%, respectively. The survival differences between the two risk groups were likely associated with *TP53* mutation status, TMB score, degree of immunocyte infiltration, and immune checkpoint level. Likewise, the expression level of every LMAG included in the model had the same effect on the overall survival and immune cell infiltration levels. More importantly, the prognostic value of the signature was verified in an independent ICGC cohort. Thus, the expression levels of LMAGs are closely related to the tumor microenvironment in HCC and may serve as promising biological indicators for prognosis and immune therapy in patients with HCC.

## Introduction

Hepatocellular carcinoma (HCC) accounts for a large proportion of cancer-related morbidity and mortality worldwide (Sayiner et al., 2019). It is the fifth leading cause of cancer-related deaths, causing 500,000 deaths annually (Bray et al., 2018). Although there has been improvement in HCC treatment modalities, including surgery, interventional therapy, and immune therapy, the prognosis remains unsatisfactory (Forner et al., 2018; Llovet et al., 2018). Therefore, biomarkers that can predict survival or treatment efficiency are urgently required. As only a small number of biomarkers are useful for predicting HCC prognosis, clinicians strive to discover new and efficient predictive parameters.

As lipids play important roles in processes regulating biological behavior, such as energy conversion, information recognition, and transmission (Xu et al., 2021), lipid metabolism is gaining global interest. Accumulating evidence has illustrated that lipid metabolism is closely correlated with chronic aging diseases (Partridge et al., 2018), cancer proliferation, and metastasis (Currie et al., 2013; Beloribi-Djefaflia et al., 2016). In particular, fatty acid synthase (FASN) is highly expressed in highly metastatic HCC cells, and suppression of FASN leads to inhibition of HCC metastasis (Hao et al., 2014). Ding et al. (2022) pointed out that the acidic tumor microenvironment activates the PI3K/AKT signaling pathway and promotes SCD1-PPARα, which causes lipid accumulation during liver cancer and promotes liver tumorigenesis (Ding et al., 2022). Therefore, lipid metabolism may play a pivotal role in HCC tumorigenesis and progression.

The tumor immune microenvironment (TIME) has been proven to guide abnormal tissue function and plays a pivotal role in the evolution of various tumors (Roma-Rodrigues et al., 2019). The TIME consists of stromal cells, immune cells, and an external matrix (Chen et al., 2015). Tumor infiltration of immune cells is correlated with tumor progression, treatment responses, and prognoses in cancers such as breast cancer (Luen et al., 2017), neuroblastoma (Vanichapol et al., 2018), and HCC (Ding et al., 2018). Taken together, the TIME has been proven to play a prominent role in tumorigenesis and tumor progression. Exploring the TIME in HCC could provide insights for improving the prognosis and efficiency of immune therapy for HCC.

In this study, we attempted to determine the effects of the expression levels of lipid metabolism-associated genes (LMAGs) on the prognosis of patients with HCC. Moreover, a close association between LMAGs and the TIME in HCC was discovered. A risk model based on four differentially expressed LMAGs was constructed, which could serve as an indicator of HCC prognosis. Our study sheds light on the roles of LMAGs in HCC and provides information that may help clinicians improve patient prognosis and develop personalized treatment regimens for HCC.

## Methods

### Data collection and identification of lipid metabolism-associated genes

The RNA-sequencing (RNA-seq) data and corresponding clinical information of 371 HCC and 50 normal patients up to 15 January 2022, were downloaded from TCGA. Additionally, the RNA-seq data and clinical information of 231 patients with HCC were retrieved from the Internal Cancer Genome Consortium (ICGC) database. All samples were the specimen bulk RNA from TCGA and ICGC databases. Additionally, the corresponding somatic mutation data were collected from the TCGA database. Samples with missing information on status, TMN stage, or overall survival (OS) were excluded. The information on a total of 491 LMAGs was obtained from the MSigDB and KEGG databases for subsequent analysis.

### Identification of lipid metabolism-associated gene subgroups by consensus clustering

Consensus clustering of LMAGs based on gene expression profiles of HCC samples was performed using the ConsensusClusterPlus package in R. For maximal stability, cumulative distribution function plots were generated for each k to obtain the optimal k value (k = 2).

### Generation of a prognostic risk model based on lipid metabolism-associated genes in hepatocellular carcinoma

Univariate Cox regression analysis was utilized to investigate the relationship between the OS and LMAG expression levels in patients with HCC. Thereafter, to minimize the risk of overfitting, LASSO Cox regression analysis was performed with the “glmnet” R package. Eventually, the expression levels of LMAGs validated by LASSO analysis were used to generate a multivariate Cox regression model based on a two-step method. The final LMAGs and their coefficients were verified, and the risk score of each HCC sample was calculated using the following equation: risk score = β1 × Exp1 + β2 × Exp2 + βi × Expi, where Expi represents LMAG expression in HCC and βi represents its coefficient. The median risk score estimated by this equation was used to stratify patients with HCC, whose samples were retrieved from TCGA, into high- and low-risk groups.

### Immune cell infiltration and tumor mutation burden analyses

The single-sample gene set enrichment analysis (ssGSEA) method was used to analyze the immunocyte infiltration and immune-related functions *via* the “gsva” R package. This analysis showed the relative degree of infiltration of each immune cell type in each sample. Additionally, TIMER was used to assess the abundance of six infiltrating immune cell types. The somatic mutation profiles of the HCC samples were obtained using the VarScan platform in the TCGA-HCC cohort. Thereafter, the “Maftools” package in R was utilized to analyze the quantity and quality of gene mutations. Moreover, the effect of LMAG expression combined with TMB scores on prognosis was investigated.

### Statistical analysis

The Kaplan–Meier survival method and log-rank test were used to evaluate the OS rate. Receiver operating characteristic (ROC) curves were generated to estimate the accuracy of the prognostic model. The chi-squared test was used to analyze the distribution of age, sex, tumor grade, tumor stage, and survival status between the two subgroups. Statistical significance was set at *p* < 0.05**.**

## Results

### Consensus clustering identified two lipid metabolism-associated gene subgroups in hepatocellular carcinoma

**FIGURE 1:**
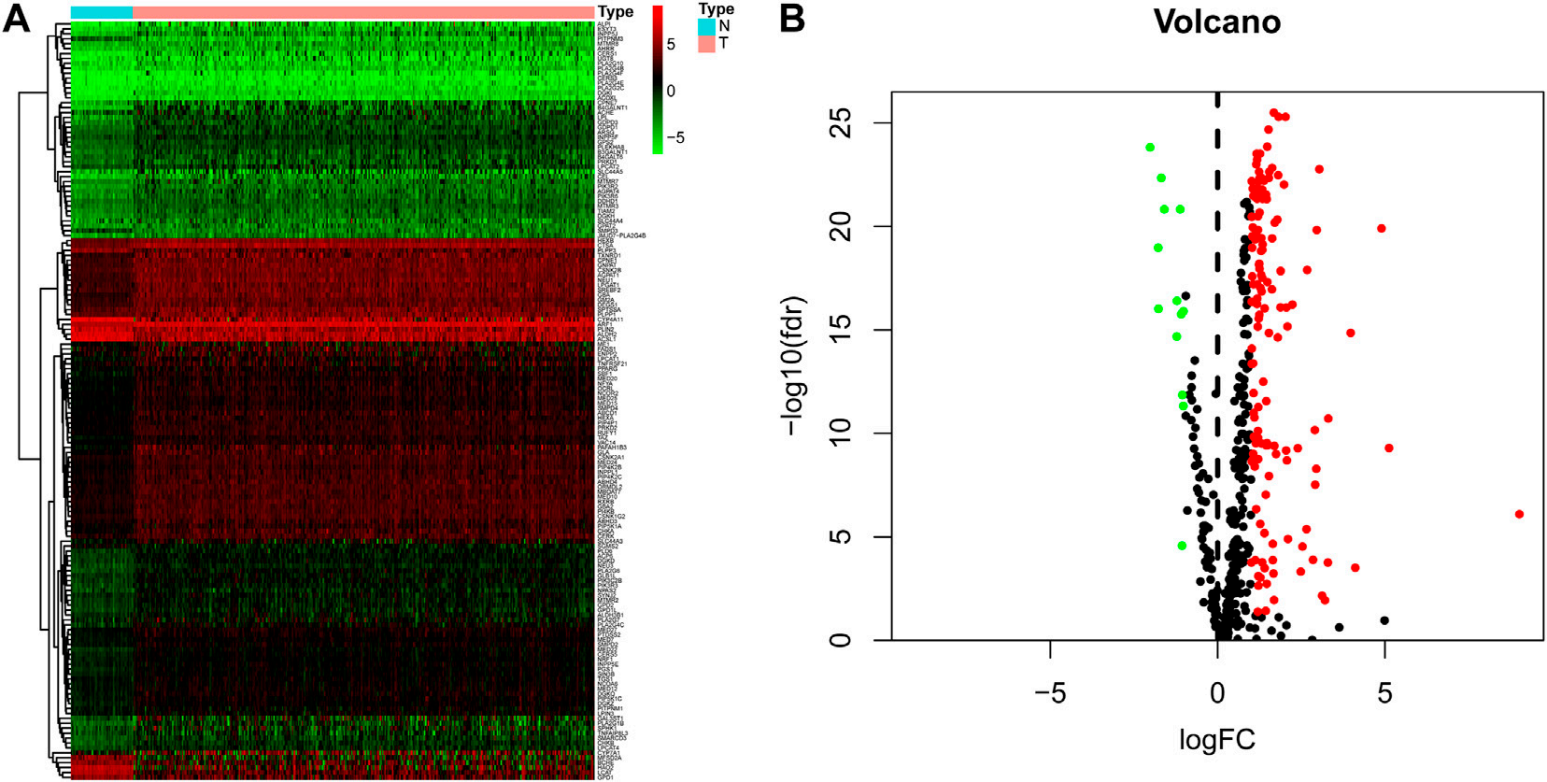
Differentially expressed lipid metabolism-associated genes in hepatocellular carcinoma (HCC) patients. Heatmap **(A)** and volcano plot **(B)** demonstrate differentially expressed lipid metabolism-associated genes between HCC and non-tumor tissues. Red and green tones represent up-regulated and down-regulated expression levels, respectively.

**FIGURE 2:**
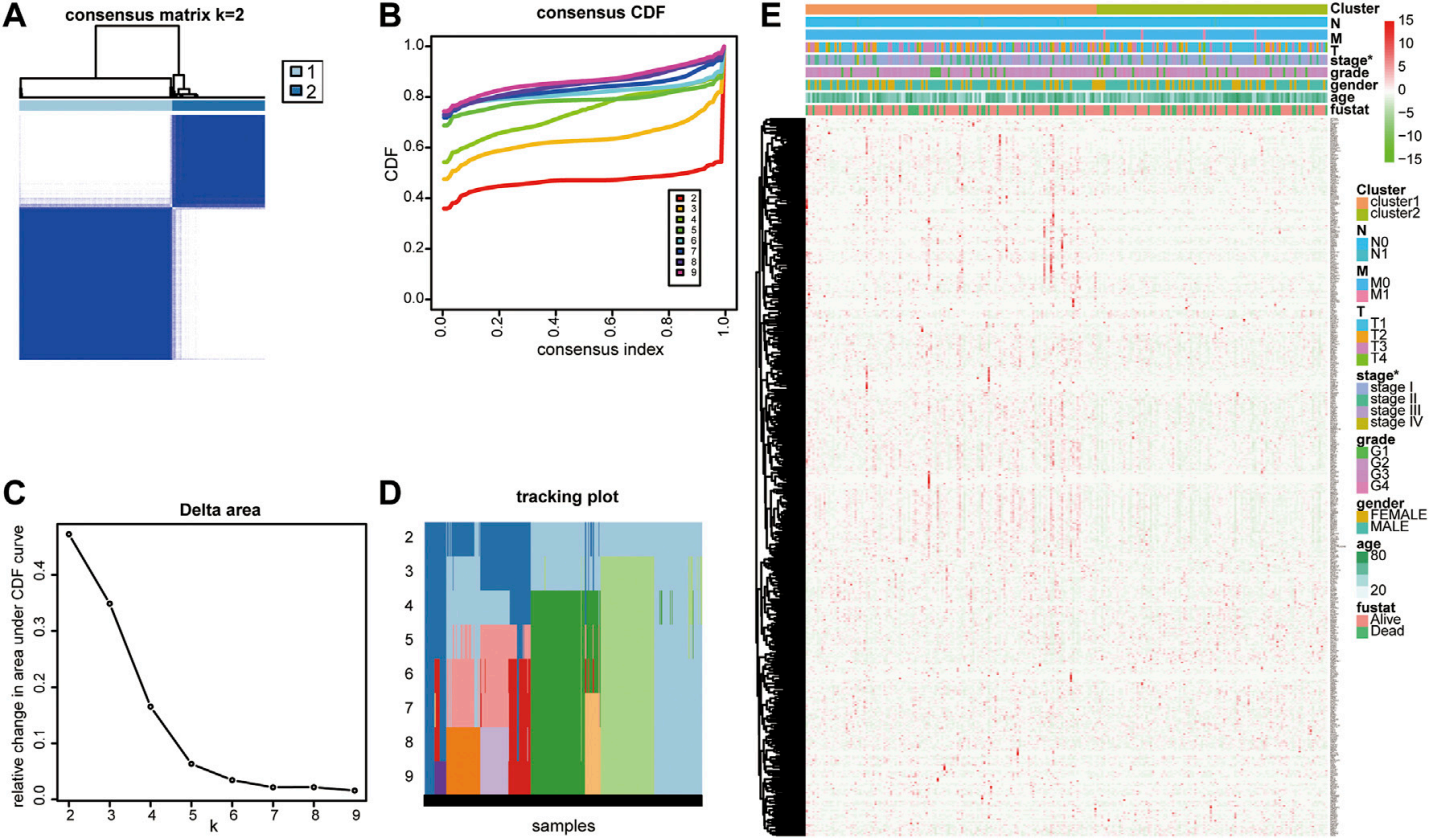
Consensus clustering identified two lipid metabolism-associated gene subgroups. **(A)** Consensus clustering matrix for k = 2. **(B)** Consensus clustering cumulative distribution function (CDF), **(C)** the relative change of area under the CDF curve and **(D)** tracking plot from k = 2 to 9. **(E)** Heatmap of correlation between lipid metabolism-associated genes with clinicopathological characteristics of HCC patients.

On comparing 371 tumor and 50 normal tissues from the TCGA database, 154 differentially expressed LMAGs (141 upregulated and 13 downregulated in tumor tissues) were identified (Figure 1; Supplementary Table S1), which indicated that LMAGs might play a role in the biological behavior of HCC. The value of k was optimized based on maximal clustering stability and set as 2. Thereafter, HCC samples were divided into cluster 1 and cluster 2 (Figures 2A–D). Upon comparing the clinical features of these clusters, cluster 2 was identified to be more closely associated with later tumor stages than cluster 1 (Figure 2E).

### Lipid metabolism regulators were associated with immune cell infiltration

**FIGURE 3:**
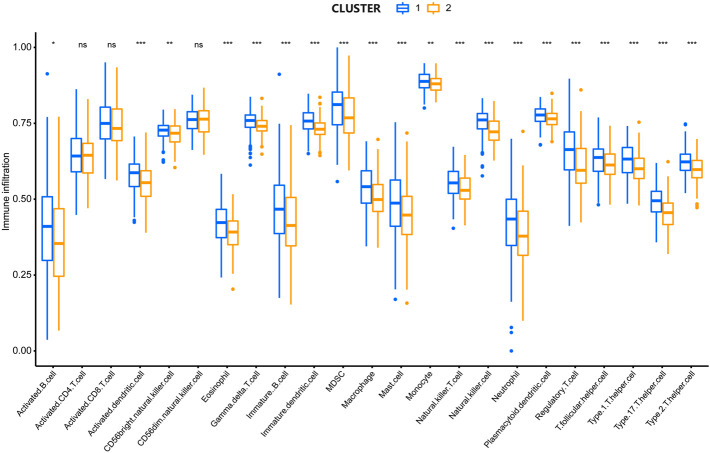
Comparison in the degree of immune cell infiltration between two clusters. *P < 0.05, **P < 0.01, ***P < 0.001.

To analyze the association between lipid metabolism regulators and the TIME in HCC, we calculated the degree of immune cell infiltration between the two clusters using ssGSEA. Cluster 1 showed higher levels of activated B cells, activated dendritic cells, CD56^bright^ natural killer cells, eosinophils, gamma delta T cells, immature B cells, immature dendritic cells, MDSCs, macrophages, mast cells, monocytes, natural killer T cells, natural killer cells, neutrophils, plasmacytoid dendritic cells, regulatory T cells, T follicular helper cells, type 1 helper cells, type 17 helper cells, and type 2 helper cells (Figure 3). These results illustrate that lipid metabolism regulators are closely correlated with immune cell infiltration in HCC.

### The prognostic model based on lipid metabolism-associated genes in hepatocellular carcinoma indicated a good prognostic value

**FIGURE 4:**
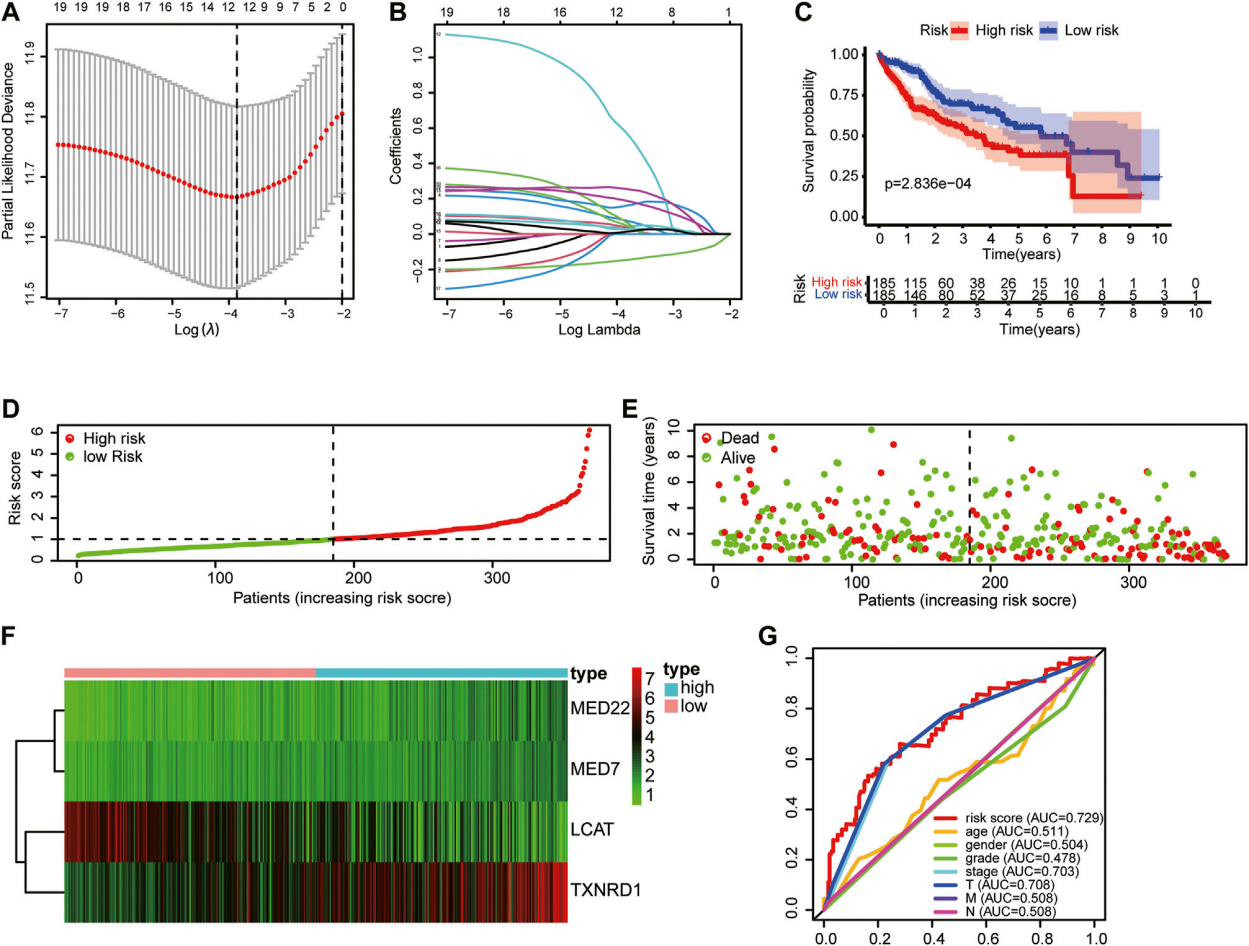
Construction of the HCC prognostic risk model based on 4 lipid metabolism-associated genes. **(A)** Lasso Cox regression analysis of lipid metabolism-associated genes. **(B)** Partial likelihood deviation for different number of variables. **(C)** Kaplan—Meier curve of OS of high-risk and low-risk HCC patients. **(D,E)** Distribution of risk score, survival status of HCC patients and heatmap of the 4 lipid metabolism-associated genes in the high/low-risk group. **(F)** The expression matrix of 4 LMAGs included in the risk model. **(G)** Receiver operating characteristic (ROC) curves of risk scores, age, gender, tumor grade and tumor stage.

Univariate Cox regression analysis identified 78 prognostic LMAGs, of which eight were validated by LASSO regression, and their data were employed to generate a multivariate Cox regression model. Finally, four prognosis-related LMAGs (mediator complex protein 22 (MED22), mediator complex protein 7 (MED7), TXNRD1, and lecithin−cholesterol acyltransferase (LCAT)) were identified, and the risk score was calculated (risk score = 0.3402 × ExpMED22 + 0.4330 × ExpMED7+ 0.2259 × ExpTXNRD1 - 0.1748 × ExpLCAT) for each HCC sample (Figures 4A,B). Based on this equation, patients whose sample data were retrieved from TCGA were classified into high- (*n* = 185) and low-risk (*n* = 185) groups. Kaplan–Meier curves demonstrated that patients in the high-risk group had poorer OS than those in the low-risk group (Figure 4C). The scatter diagram in Figure 4 shows the survival rates of patients in the high- and low-risk groups. Patients with high risk scores had a higher probability of early death than those with a low risk score (Figures 4D,E). The heatmap in Figure 4F clearly shows that the four prognostic LMAGs in the two risk groups had notably different expression levels. Thereafter, the predictive accuracy of the risk score was assessed *via* ROC analysis. The AUC values were 0.729 for risk score, 0.551 for age, 0.504 for gender, 0.478 for tumor grade, 0.703 for tumor stage, and 0.708, 0.508, and 0.508 for T-, N-, and M-staging, respectively (Figure 4G). These findings reveal that the risk score based on LMAGs in HCC has a higher prognostic value than other clinical characteristics.

### The risk model based on lipid metabolism-associated genes was an independent prognostic indicator of hepatocellular carcinoma

**FIGURE 5:**
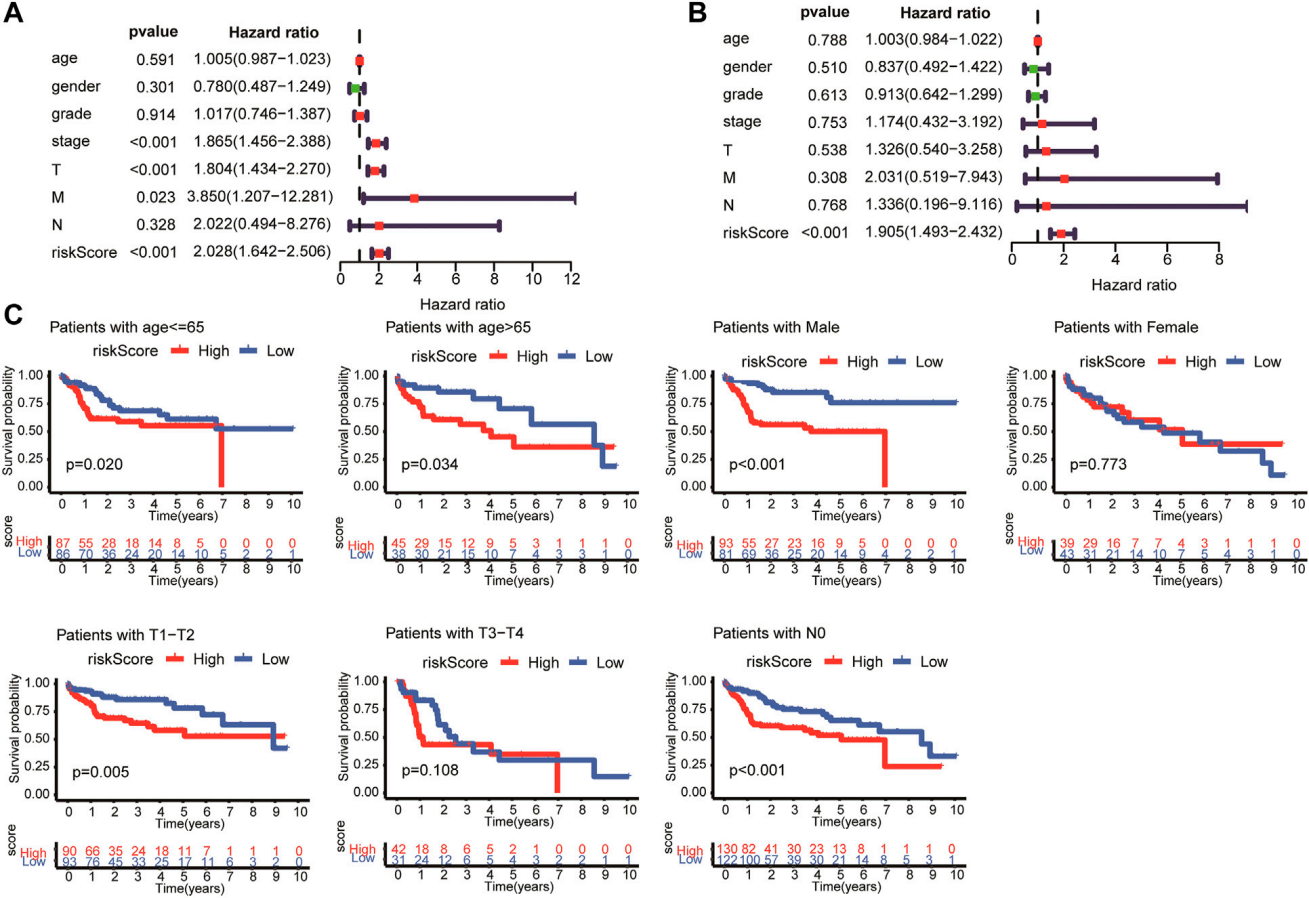
Univariate and multivariate analysis showed the risk score calculated by LASSO model was an independent prognostic predictor. Univariate **(A)** and multivariate **(B)** Cox analysis in HCC patients. **(C)** Subgroup Kaplan-Meier analysis of HCC patients in different age, sex, and tumor staging subgroups.

Univariate and multivariate Cox regression analyses were performed to determine whether the risk model based on the four LMAGs was an independent prognostic indicator of HCC. After removing cases with incomplete information, 235 participants were subjected to regression analysis. The univariate Cox regression analysis demonstrated that OS was significantly related to tumor stage (HR, 1.865; 95% CI, 1.456–2.388; and *p* < 0.01), T-staging (HR, 1.804; 95% CI, 1.434–2.270; and *p* < 0.01), M-staging (HR, 3.850; 95% CI, 1.207–12.281; and *p* = 0.023), and risk score (HR, 2.028; 95% CI, 1.642–2.506; and *p* < 0.01), as shown in Figure 5A. After further verification using multivariate Cox regression analysis, the risk score (hazard ratio [HR], 1.905; 95% CI, 1.493–2.432; and *p* < 0.01) remained the only predictive parameter for poor prognosis (Figure 5B). Furthermore, in the subgroup analysis, the patients were stratified into different subgroups according to age, sex, and tumor staging. The risk model still presented a noteworthy prognostic value, and the survival rate in the low-risk group was higher than that in the high-risk group (Figure 5C).

### The prognostic risk model correlated with the clinicopathological characteristics of hepatocellular carcinoma

**FIGURE 6:**
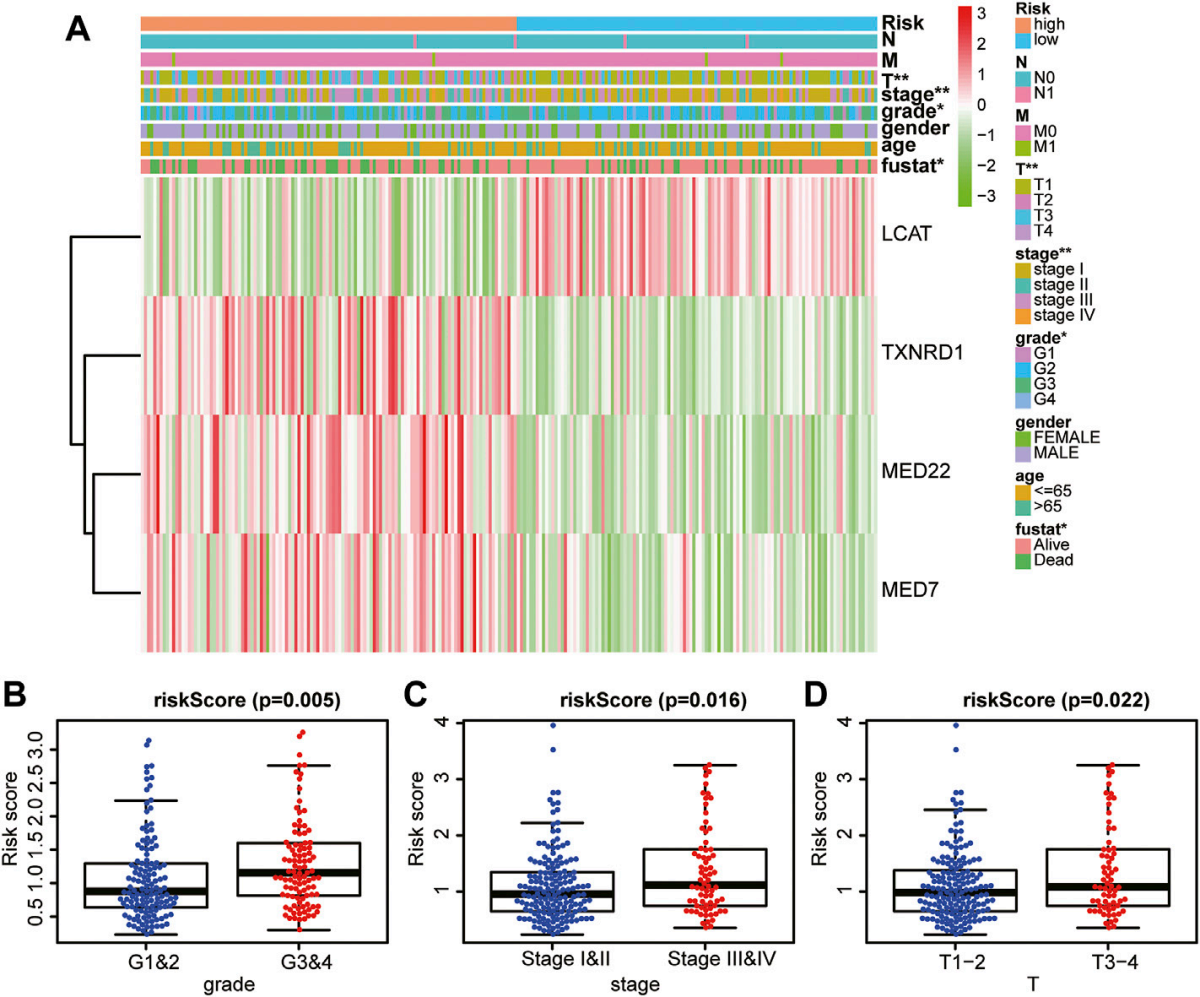
Prognostic risk score correlated with clinicopathological characteristics. **(A)** Heatmap of clinicopathological characteristics and different lipid metabolism-associated gene expression levels in high/low-risk groups. **(B–D)** Comparison of risk score distribution in the different sample classification of tumor grade **(B)**, tumor stage **(C)** and T staging **(D)**.

Next, we investigated the association between the risk score and clinicopathological characteristics of patients with HCC. Notably, as shown in the heatmap in Figure 6A, the four LMAGs in the high- and low-risk groups had distinct expression levels. In addition, the two risk groups showed significant differences in tumor grade (*p* = 0.005, Figure 6B), tumor stage (*p* = 0.016, Figure 6C), and T-staging (*p* = 0.022, Figure 6D). These results reveal that the risk score based on LMAGs might play a critical role in the clinical outcomes of patients with HCC.

### The risk model showed favorable prognostic accuracy for hepatocellular carcinoma cases in the Internal Cancer Genome Consortium database

**FIGURE 7:**
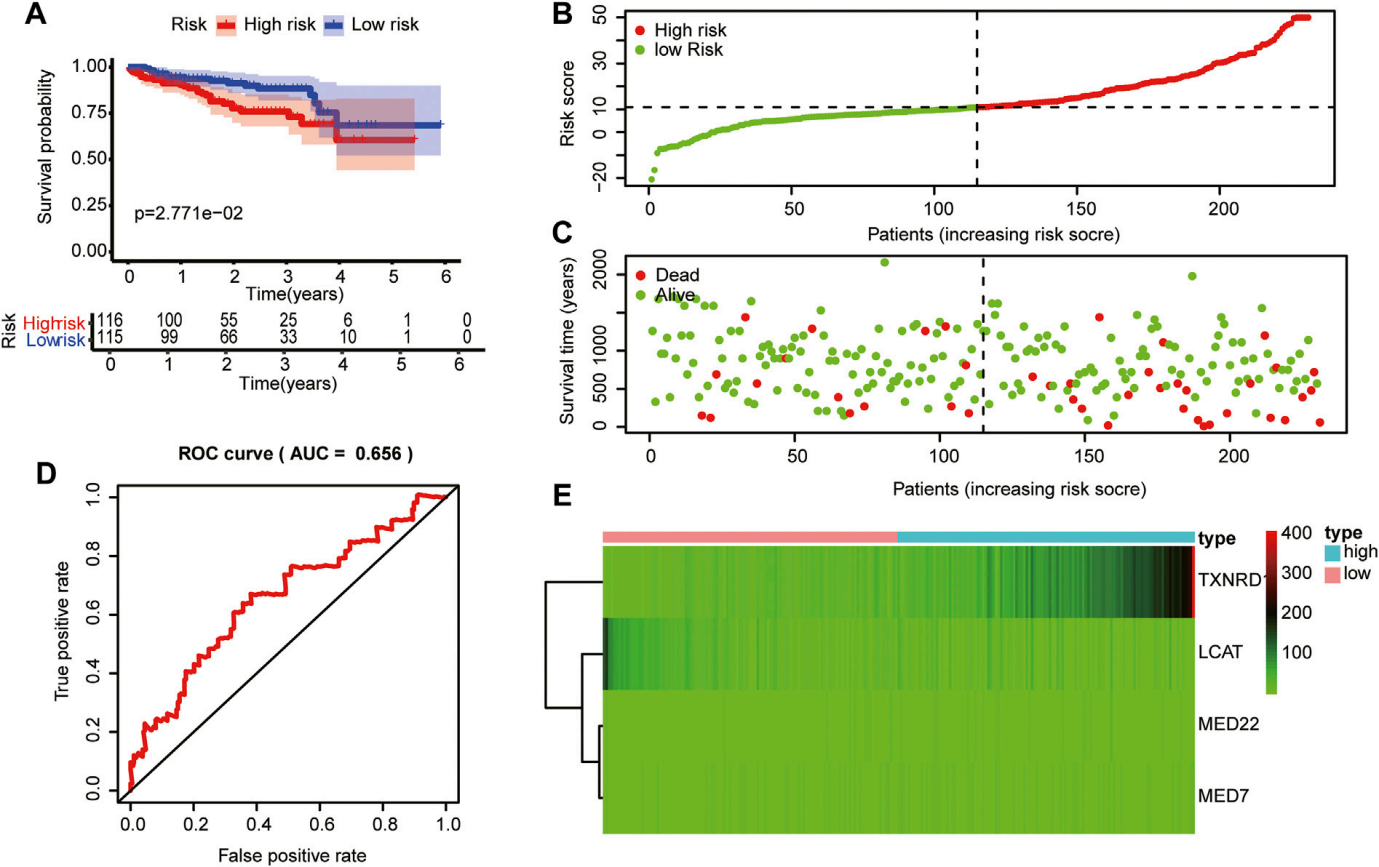
Validation of the risk model in the in the Internal Cancer Genome Consortium (ICGC) database.

To validate the prognostic value of the risk score model based on LMAG expression in other HCC cases, we collected HCC cases from the ICGC database. The risk score of each patient with HCC was calculated according to the abovementioned equation. Thereafter, the median score was used to stratify patients into high- and low-risk groups. The Kaplan–Meier curves demonstrated that patients in the high-risk group had poorer prognosis than those in the low-risk group (Figure 7A). The risk score and survival status distributions are shown in Figures 7B,C. The time-independent AUC in the ROC curve was 0.656, indicating that the constructed risk model had a favorable prognostic value (Figure 7D). The heatmap presents the expression levels of the four LMAGs, showing distinct differences between the high- and low-risk groups (Figure 7E). Consistent with the results of cases from the TCGA database, the risk model based on four LMAGs showed promising results in predicting prognosis for patient data from the ICGC database.

### Mutation profiles of different risk subgroups were explored

**FIGURE 8:**
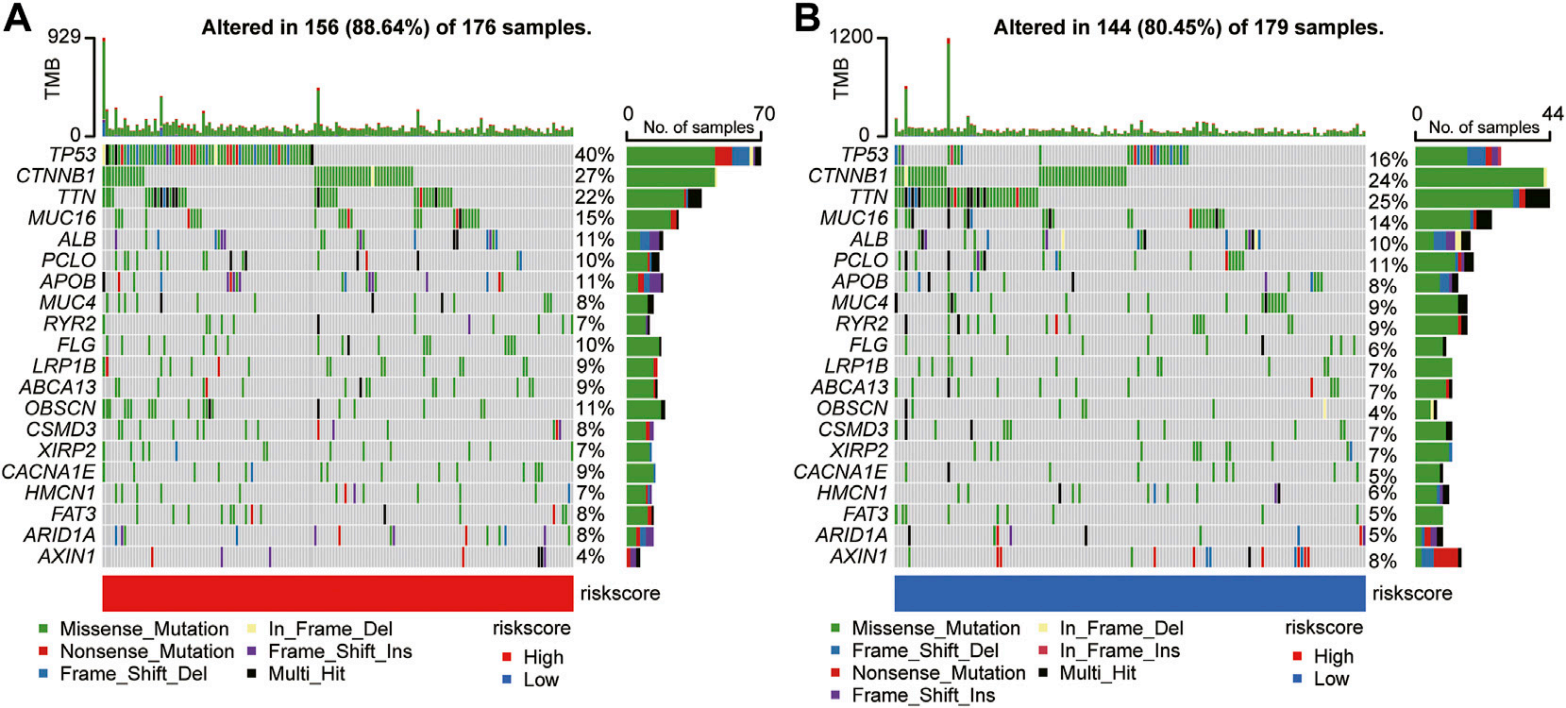
Mutational landscape of significantly mutated genes in the high-risk **(A)** versus low-risk **(B)** subgroups.

Based on an early report that gene mutation status could predict OS and immune response, the discrepancy in mutation profiles between the two risk subgroups was analyzed. Notably, the high-risk group had a higher frequency of gene mutations than the low-risk group. Figure 8 depicts the top 20 gene mutation types in the two groups, showing that missense mutations were the most frequent in HCC. In the high-risk group, mutation of *TP53* was the most common mutation type, whereas, in the low-risk group, mutation of *TNN* was more common than other mutations. The abovementioned observations may provide novel directions for exploring tumorigenesis and HCC development.

### Risk score correlated with immune characteristics in hepatocellular carcinoma

**FIGURE 9:**
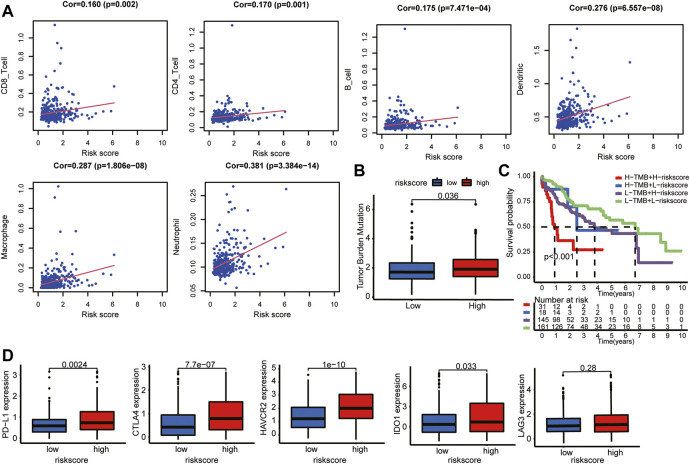
Distant immune features in two risk subgroups. **(A)** The correlation between the risk score and the immune cell infiltration. **(B)** Tumor mutation burden was compared with the two risk subgroups. **(C)** The risk model still impacted OS in the background of similar TMB scores. **(D)** Relation between risk score and expression level of immune checkpoint genes.

To evaluate the impact of the LMAG-based risk model on the TIME in HCC, we investigated the association between risk scores and immune cell infiltration levels. The risk score was positively correlated with the infiltration levels of six immune cell types, namely, CD8 T cells, CD4 T cells, B cells, dendritic cells, macrophages, and neutrophils (Figure 9A). Subsequently, the correlation between the risk scores and TMB was investigated, which revealed that the high-risk group had higher TMB levels (Figure 9B). To further illustrate the potential crosstalk between the four LMAGs and TMB, we examined whether the risk model had a significant impact on survival outcomes in the background of similar TMB scores. Our study showed that patients with high risk scores and high TMB scores had poorer survival outcomes than those with low risk scores and low TMB scores, and patients with low risk scores and low TMB scores had a better prognosis than those with high risk scores and low TMB scores (Figure 9C). When patients with high risk scores were classified based on the TMB score, the TMB score had a significant impact on the clinical outcome, and similar results were observed in the low-risk score subgroup. In addition, we observed the expression levels of immune checkpoint genes in different risk groups. Notably, the high-risk group had higher expression levels of PD-L1, CTLA4, HAVCR2, IDO1, and LAG3 (Figure 9D). Hence, we believe that the risk model based on the four LMAGs had a strong correlation with the TIME in HCC, and LMAGs, along with immune characteristics, may synergistically act as a latent prognostic signature in HCC.

### The effects of genetic alteration and expression levels of the four lipid metabolism-associated genes on the tumor microenvironment of hepatocellular carcinoma were analyzed

**FIGURE 10:**
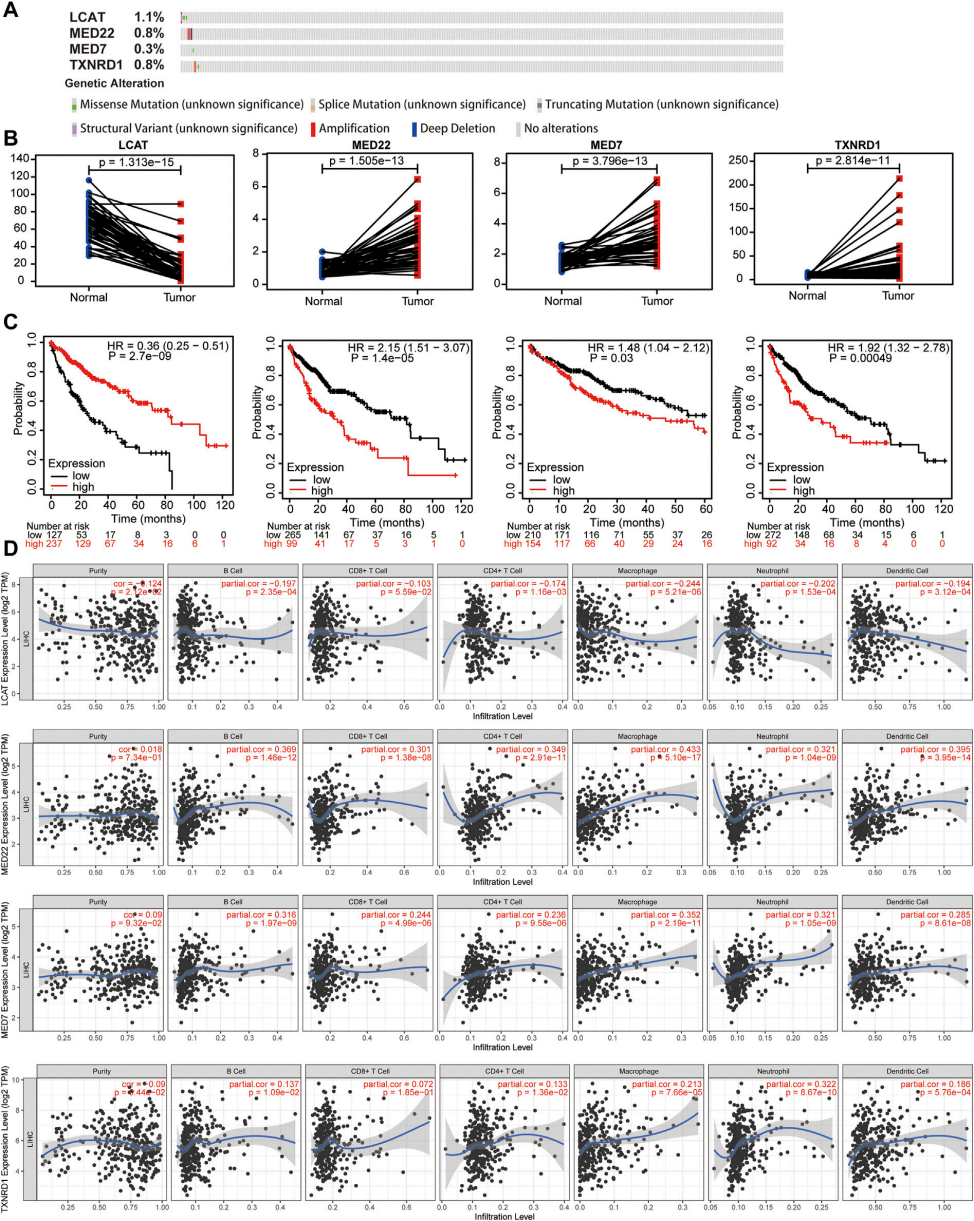
Genetic alterations, expression levels, prognosis and correlation with immune cells of the four predictive lipid metabolism-associated genes. Genetic alterations **(A)**, expression levels **(B)** and survival outcomes **(C)** of the 4 lipid metabolism-associated genes. **(D)** Effects of the expression levels of four LMAGs on the immune cell infiltration.

**FIGURE 11:**
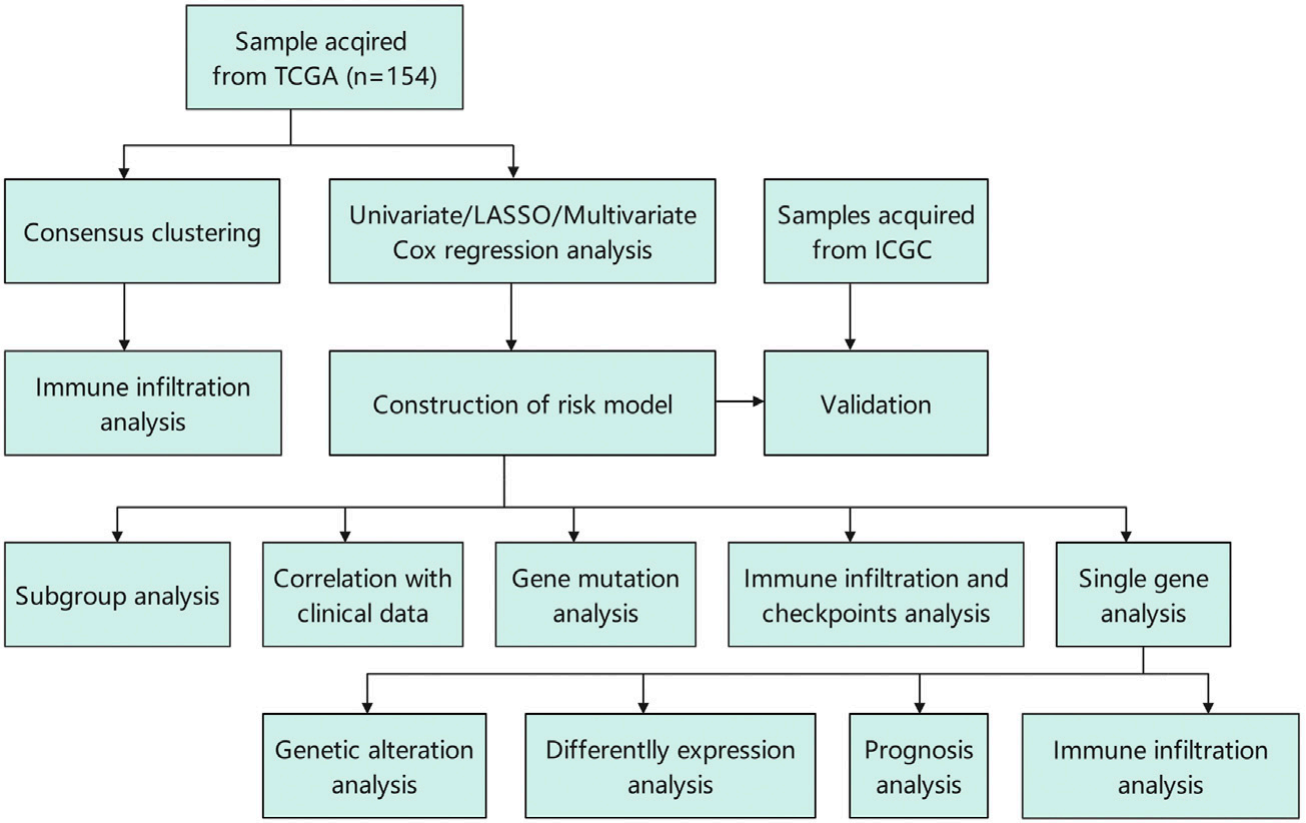
Flowchart of this study.

The genetic alteration and expression levels of the four LMAGs in HCC were analyzed. Notably, the genetic alteration levels of *LCAT* were the highest compared to other LMAGs (1.1%), and the most frequent alterations among the four LMAGs were amplification and deep deletion (Figure 10A). Moreover, the effects of expression levels of the four LMAGs on HCC prognosis were investigated. Consistent with previous observations, *LCAT* had low expression levels during HCC, whereas the other three LMAGs had higher expression levels (Figure 10B). In addition, patients with overexpressed *LCAT* and underexpressed *MED22, MED7*, and *TXNRD1* genes had better prognosis (Figure 10C). Finally, the association between the expression levels of the four LMAGs and immune cell infiltration levels was explored. As shown in Figures 10A,D, a strong correlation was observed between the immune cell infiltration and LMAG expression levels. The procedure used in this study is illustrated in Figure 11.

## Discussion

HCC is a highly heterogeneous, malignant tumor with complicated etiology. Although HCC management has continued to improve over time, high morbidity and mortality due to the disease have raised global concerns. Unfortunately, owing to insufficient prospective and validated research works, no useful biomarker has been approved for clinical practice in HCC treatment. Thus, there is an urgent need to identify efficient and complementary prognostic parameters and risk classification methods. Accumulating evidence suggests that lipid metabolism is closely related to tumorigenesis and HCC development. For example, Zhang discovered that fat storage-inducing transmembrane protein 2 (FITM2) is highly expressed in HCC tissues and could be an independent prognostic parameter of OS during HCC (Chen et al., 2021). Furthermore, several biological experimental models have established the idea that elimination of FASN delays the tumorigenesis of HCC and prevents tumor proliferation (Cao et al., 2017; Che et al., 2020). Among the drugs that affect lipid metabolism, statins are best known for lowering cholesterol levels, and some studies have demonstrated that atorvastatin restores cholesterol-induced gut microbiota dysbiosis and completely prevents NAFLD-HCC development (Zhang et al., 2021). Given that previous studies have made efforts to illustrate how lipid metabolism is reprogrammed during HCC, further research focusing on the relationship between lipid metabolism and HCC prognosis and the immune microenvironment is urgently required.

First, our study revealed that LMAGs were differentially expressed in HCC tissues compared to normal tissues and were closely correlated with immune cell infiltration. A previous study reported that a microenvironment created by crosstalk between tumor cells and adjacent immunocytes was capable of regulating tumor development and metastasis (Hinshaw and Shevde, 2019). Thus, we speculated that lipid metabolism contributed to the TIME in HCC. Subsequently, we constructed a risk model based on four LMAGs—*LCAT*, *MED22*, *MED7*, and *TXNRD1*—which presented noteworthy prognostic values and was closely associated with tumor grade, tumor stage, and T-staging. Consistent results were obtained using the data from the ICGC database.

The four LMAGs employed in our risk model are validated pivotal factors in cancer development. LCAT has been associated with breast cancer (Subbaiah et al., 1997), ovarian cancer (Russell et al., 2017), and Hodgkin’s lymphoma (Cooke et al., 2018). Consistent with the previous finding that LCAT is less active in patients with liver diseases (Tahara et al., 1993), our study demonstrates that LCAT is underexpressed in HCC. A study pointed out that overexpression of LCAT could increase plasma levels of HDL, leading to a more efficient reverse cholesterol transport (Seguret-Mace et al., 1996). In liver diseases, the underexpression of LCAT may result in restricted cellular cholesterol efflux, which increases the risk for fatty liver disease and liver cancer. MED7 and MED22 are subunits of the mediator complex (MED). MED proteins play a key role in eukaryotic transcription (Malik and Roeder, 2010) by regulating the ability of transcription factors to transfer information to the polymerase II-transcription machinery (Weber and Garabedian, 2018). With respect to other tumors, MED7 underexpression was correlated with an increased risk of gastrointestinal stroma (Koschubs et al., 2009), while in breast cancer, especially ER + luminal subtypes, MED7 was positively correlated with improved prognosis (Joseph et al., 2018). Moreover, MED22 was found to be significantly correlated with prognosis of HCC (Wang et al., 2021). With respect to TXNRD1, the fourth LMAG identified in the study, inhibiting the NRF2/TXNRD1 signaling axis has been demonstrated as an efficient approach to restrain HCC growth, and combination therapy with sorafenib and NRF2/TXNRD1 inhibitors may be a promising strategy for personalized HCC treatment (Gao et al., 2020). Taken together, the risk model presented in our study has high prognostic value and provides innovative insights into the possible roles of LMAGs in HCC progression. This might be an important translational point in guiding the prescription of drugs affecting lipid metabolism, such as statins. Future studies should focus on the relationship between LMAGs and HCC-targeted drugs such as sorafenib.

The TIME, formed by malignant tumor cells and proximal immune cells, reveals the biological course of the antitumor immune response. Therefore, it is considered a crucial factor in the relationship of malignancy with the degree of immune cell infiltration and immune response (Anderson et al., 2006). Given that the risk model constructed using LMAGs had a high prognostic value, we aimed to discover whether LMAGs were related to the TIME in HCC. First, the gene mutation statuses of the two subgroups were determined. Missense mutations were the most frequent mutation type. In the high-risk group, *TP53* mutations were the most common, while in the low-risk group, *TNN* mutations were the most common among all types. These findings indicate that the gene mutation data for HCC are distinct between the high- and low-risk groups. Tumor immune infiltration analysis using TIMER showed that the high-risk group had higher degrees of infiltration for all six immune cell types, namely, CD8 T cells, CD4 T cells, B cells, dendritic cells, macrophages, and neutrophils. Thereafter, we measured TMB, a prognostic biomarker for immune checkpoint therapy, in various tumor types (Romero, 2019). Unsurprisingly, the high-risk group exhibited high TMB levels, and in the background of similar TMB degrees, the risk score still acted as a predictive parameter of clinical outcome. Additionally, the expression of the immune checkpoint genes *PD-L1, CTLA4, HAVCR2, IDO1*, and *LAG3* was dramatically upregulated in the high-risk subgroup. Immune checkpoint inhibitor therapy, such as anti-PD-L1 antibody therapy, has been shown to be of high clinical value in various malignancies (Chinai et al., 2015). A clinical trial has shown that the direct targeting of LAG3 together with anti-PD-1 antibody administration induces persistent immune responses in patients with previously untreated, unresectable, or metastatic NSCLC (NCT03625323). Recently, the combination of two cycles of platinum-based chemotherapy with the anti-PD-1 agent nivolumab and the anti-CTLA-4 agent ipilimumab achieved improved outcomes compared to first-line chemotherapy in a randomized phase III CheckMate 9LA trial (Genova et al., 2021). Our results suggest that LMAGs may play an instructive role in immune checkpoint inhibitor therapy for HCC. Although the current gold-standard treatment for HCC is the use of the imBRAVE150 regimen involving bevacizumab and azazolizumab, it was difficult to validate the effectiveness of this protocol in our study; hence, its supreme efficacy remains a theory, and more experiments and trials are required in this respect. Finally, gene mutation levels, expression levels, prognostic values, and the effects on immune cell infiltration of the four LMAGs included in our risk model were analyzed. Consistent with the abovementioned observations and further validating our theory, significant associations of the expression levels of the four LMAGs with the clinical outcomes and immune cell infiltration were identified. In line with previous findings, it is reasonable to assume that the regulation of lipid metabolism might lead to impairment of the TIME in HCC. Hence, targeting lipid metabolism pathways can be a promising method for inducing an immune response in HCC treatment.

Nevertheless, this study has some potential drawbacks that need to be addressed. First, the proposed risk model based on four LMAGs was verified using only a limited amount of data obtained from TCGA and ICGC databases. Further validation using other external cohorts and clinical cases is required to test the precision of our risk model. Next, our study was based on bioinformatics analysis, and the effect of LMAGs on the TIME of HCC requires experimental verification *in vivo* and *in vitro*. Third, given the low number of clinical samples, it was difficult to confirm the correlation of LMAGs with lipid metabolism-targeted and chemotherapeutic drugs. Finally, we did not study the interactions between LMAGs in the TIME; future studies focusing on these interactions are needed to remodel the tumor microenvironment and enhance the efficiency of HCC immunotherapy.

## Conclusion

We performed a systematic analysis of the regulatory functions of LMAGs in HCC and their effect on prognosis. The risk model identified four LMAGs as independent prognostic factors for HCC and was closely related to immune characteristics and immune response. This study sheds light on the potential of LMAGs as indicators of HCC prognosis and individualized immune therapy.

## Data Availability

The datasets for this study can be made available by the authors without undue reservation.
