# Supplementary figures and images for: Identification of lipid metabolism-associated genes as prognostic biomarkers based on the immune microenvironment in hepatocellular carcinoma
Source: Front Cell Dev Biol. 2022 Oct 18;10:883059. doi: 10.3389/fcell.2022.883059 (PMC9622944; doi:10.3389/fcell.2022.883059)

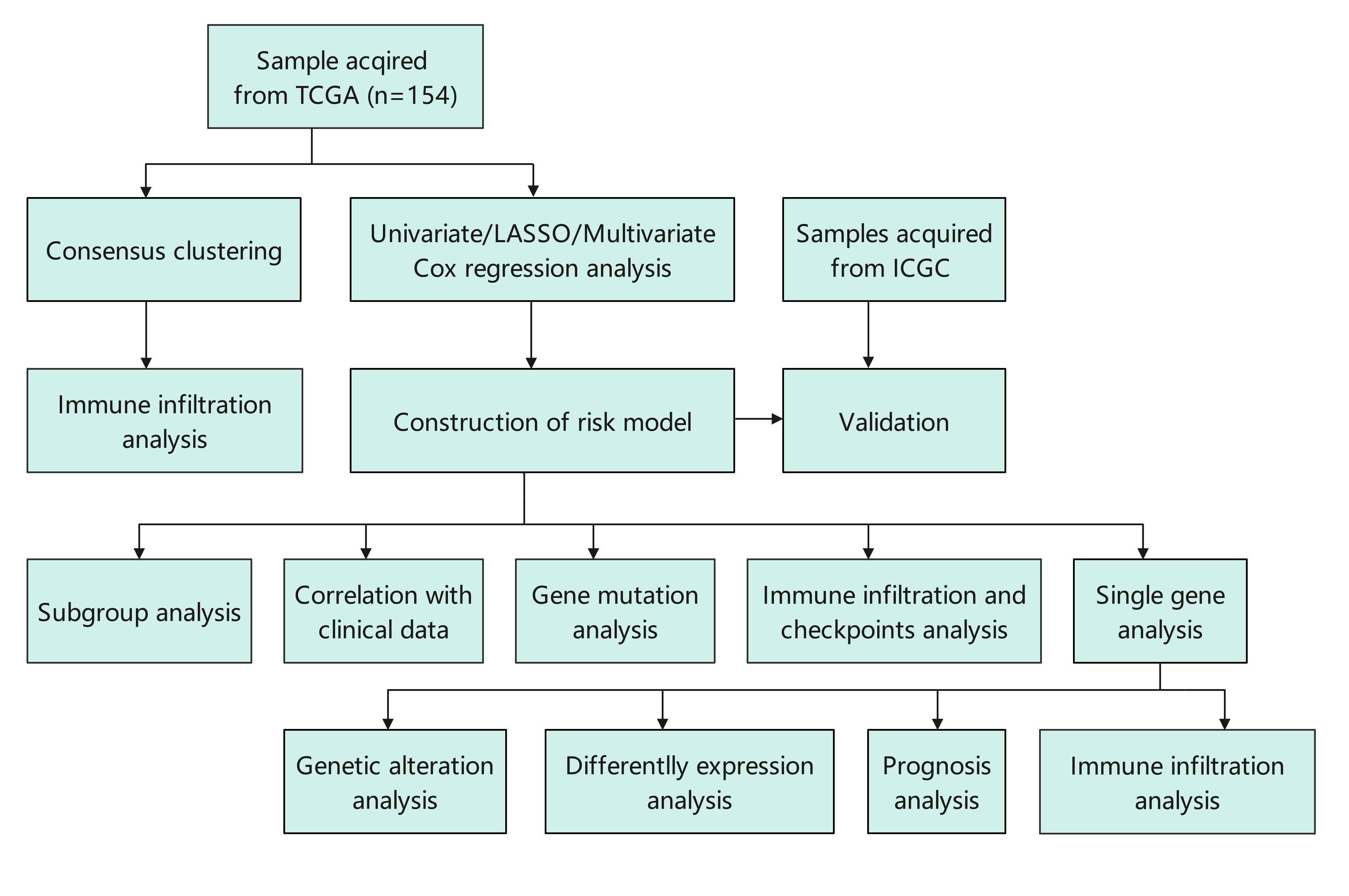

Supplement: Supplementary file 1 [file Image1.JPEG]
